# Pea Protein and Maltodextrin‐Based Encapsulation of Anthocyanins From Black Rice Bran: Characterization, Storage Stability, and In Vitro Anthocyanin Release

**DOI:** 10.1111/1750-3841.71184

**Published:** 2026-06-18

**Authors:** Eduardo Leonarski, Gabriela Polmann, Guilherme Dallarmi Sorita, Paulo Alexandre Durant Moraes, Karina Cesca, Débora de Oliveira, Acácio Antonio Ferreira Zielinski

**Affiliations:** ^1^ Department of Chemical Engineering and Food Engineering Federal University of Santa Catarina (UFSC) Florianópolis Brazil; ^2^ Department of Chemistry Federal University of Santa Catarina (UFSC) Florianópolis Brazil

**Keywords:** by‐product, cyanidin 3‐glucoside, in vitro release, *Oryza sativa* L., stability

## Abstract

This study aimed to encapsulate anthocyanins from black rice bran (an agro‐industrial by‐product) using pea protein (PR) and maltodextrin (MA) as wall materials. The microcapsules were characterized by determining encapsulation efficiency, structural and thermal characteristics, color and storage stability, and in vitro anthocyanin release. Two formulations were prepared, both containing 2.5% anthocyanin (ANC), one containing 100% PR (PP) and the other containing 50% PR and 50% MA (PM). The encapsulation efficiency (EE) was higher for PM (94.7%) than for PP (90.2%). Furthermore, the particle size was 2.2‐fold higher for PM due to the addition of MA. After 60 days, the stability of both microcapsules was significantly higher (approximately 66%–70%) than that of ANC (54%) and did not differ significantly between them (*p* > 0.05). Regarding the evaluation of anthocyanin release, the PM sample in the gastric phase presented a release of 34.7%. In comparison, PP was 29.2%, but at the end of the intestinal phase, the PP sample presented a total of 56.2% anthocyanin release, while PM presented 59.6%, differing from each other (*p* < 0.05). Overall, the addition of PR was essential to increase the storage stability of anthocyanins through encapsulation, presenting high EE and good release during the in vitro digestibility of these compounds. Microcapsules have the potential to be applied in several areas, especially in the food industry.

**Practical Applications:**

The key contribution of this research is that the food and supplement industries can use microcapsules as a natural, stable coloring agent and antioxidant booster derived from rice waste. They protect sensitive nutrients during storage and ensure their controlled release in the body, offering a healthier, plant‐based alternative to synthetic dyes in functional foods.

## 1 Introduction

Anthocyanins are water‐soluble natural colorants belonging to the group of flavonoids that have bioactive properties due to their antioxidant properties (Luzardo‐Ocampo et al. 2021; Tarone et al. 2020). Anthocyanins are found naturally in fruits, vegetables, flowers, leaves, cereals, grains, roots, and tubers (Arruda et al. 2021; Tena et al. 2020). With market demand for replacing synthetic colorants with natural colorants, industries are moving to meet this requirement, with natural colorants serving as alternatives to this expectation, offering good color properties and several health benefits (Philippini et al. 2020; Sanna and Fadda 2022). According to Meticulous Research (2024), the natural dye market is expected to reach $3.74 billion by 2031, with a compound annual growth rate (CAGR) of 6.8% from 2024 to 2031.

A substantial portion of natural colorants (anthocyanins, phycobiliproteins, betalains, carotenoids, and chlorophylls) can be extracted from agro‐industrial by‐products, representing a viable way to upcycle these materials that would otherwise be discarded (Magalhães et al. 2024; Nutrizio et al. 2024). During the processing of black rice, the main solid by‐products generated include straw, husk, bran, and broken rice. Bran and broken rice are rich in anthocyanins and can be used as a source for the recovery of these bioactive compounds (Leonarski, Kuasnei, Cesca, et al. 2024). However, these compounds offer low stability mainly due to sensitivity to pH, light, oxygen, temperature, and other substances (enzymes, pigments, and metal ions) (Castañeda‐Ovando et al. 2009; Yang et al. 2020). Therefore, for anthocyanins to be widely used, it is necessary to compensate for these deficiencies to maintain coloration and health‐beneficial properties (Yang et al. 2020).

Some methods have been used to improve the stability of anthocyanins, such as encapsulation by freeze‐drying (Tan et al. 2024; Mansour et al. 2020) or spray‐drying (B. Li, Zhao, et al. 2022; Norkaew et al. 2019), copigmentation (Lv et al. 2022; Gençdağ et al. 2022), and acylation (Leonarski, Cesca, et al. 2025; Marquez‐Rodriguez et al. 2021). Encapsulation also offers numerous advantages for bioactive chemicals, including improved oxidative, thermal, and photochemical stability, bioavailability, taste masking, sustained and regulated release, and greater ease of handling (Fu et al. 2024).

Natural nanostructured polymers, such as polysaccharides, proteins, and lipids, are widely used as encapsulating agents for anthocyanins due to their low toxicity, chemical characteristics, and mechanical strength (Yin et al. 2022). Proteins from animal origin (whey protein or casein) are generally used as wall material, although several proteins of plant origin (mainly from legumes) have been widely developed in recent years, such as pea protein (Shanthakumar et al. 2022; J. Wang et al. 2022), dry beans (Ferreira et al. 2022; Teixeira et al. 2023), chickpeas (Grasso et al. 2022; Boukid 2021), lentils, and mung beans (Shrestha et al. 2023; Miranda et al. 2022; Huang et al. 2024).

Pea protein is characterized by two main globulin proteins: legumin and vicilin (Muñoz‐Coyotecatl et al. 2025). Quercetin was encapsulated with pea protein and high‐methoxyl pectin, and its thermal stability and antioxidant properties significantly improved (J. Li, Zhang, et al. 2022). The authors also observed that using pH 6.0 (compared to pH 5.0) resulted in a greater protective effect and a higher percentage of quercetin release in the simulated gastrointestinal fluid.

Therefore, our study aimed to extract and purify anthocyanins from black rice bran (BRB). Furthermore, the extracts were encapsulated using a combination of pea protein and maltodextrin as wall material. To the best of our knowledge, this is a study with relevant results concerning the encapsulation of BRB anthocyanins using pea protein as the wall material.

## 2 Materials and Methods

### 2.1 Extraction and Purification of Anthocyanins From BRB

BRB (supplied by Ruzene, Pindamonhangaba, Brazil) was ground in a Wiley mill (DeLeo, Brazil) and sieved (particle size between 20 and 32 mesh). Anthocyanin extraction was performed using an ultrasound probe (Eco‐Sonics, Ultronique QR550, Brazil). In a glass reactor, 5 g of BRB was used for 300 mL of solvent (40:60, ethanol:0.1 M citric acid). The process parameters used were as follows: 5 min (defined by kinetics), temperature of 50°C, and frequency power of 450 W. After extraction, the extracts were centrifuged, and the ethanol was evaporated using a rotary evaporator (Leonarski, Kuasnei, Santos, et al. 2024; Leonarski, Cesca, et al. 2025).

For purification, the concentrated extract (approximately 100 mL) was subjected to liquid‒liquid extraction three times with ethyl acetate (1:1), and the extract was evaporated again. The aqueous fraction was concentrated and purified in a Buchner funnel loaded with SPE DSC‐18 silica gel (Sigma‐Aldrich, Darmstadt, Germany). The system was washed with 5 L of deionized water (0.01 M HCl). Then, a 50:50 (v/v) water/ethanol fraction was acidified with 0.01 M HCl to elute the anthocyanins. Subsequently, the ethanol was rotary evaporated, and the extract was lyophilized.

### 2.2 Total Monomeric Anthocyanins

The total monomeric anthocyanin (TMA) concentration was determined according to the differential pH methodology (Giusti and Wrolstad 2001). Then, 20 µL of anthocyanin‐rich extract was added to microplates to 280 µL of a buffer solution with pH 1.0 (potassium chloride 0.025 mol/L) and pH 4.5 (sodium acetate 0.4 mol/L). The absorbances were read at 520 and 700 nm (Multileader Infinite M200 TECAN, ZH, Switzerland), and the concentrations of TMA were calculated using Equations (1) and (2):

(1) $$A={\left(A_{520}-A_{700}\right)}_{\mathrm{pH}\;1.0}-{\left(A_{520}-A_{700}\right)}_{\mathrm{pH}\;4.5}$$

(2) $$\mathrm{TMA}=\frac{A\times \mathrm{MW}\times \mathrm{DF}\times 1000}{\varepsilon }$$

where *A* is the calculated absorbance, MW is the molecular weight of the anthocyanin standard (cyanidin 3‐glucoside [C3G]: 449.2 g/mol), DF is the dilution factor (15), and *ε* is the molar absorptivity of cyanidin (26,900).

### 2.3 Individual Anthocyanin by HPLC–PAD

Individual anthocyanins were evaluated by HPLC (Model LCMS‐2020 Prominence, Shimadzu, Kyoto, Japan) equipped with a photodiode array detector (PAD; SPD‐M20A) and a reversed‐phase column 250 mm × 4.6 mm i.d., 5 µm (C18‐column, PerkinElmer Brownlee Analytical N9303514), thermostated at 25°C, according to Leonarski et al. (2025). The mobile phases were (A) water/formic acid (99.5:0.5, v/v) and (B) acetonitrile/formic acid (99:1, v/v), following the gradient: 0%–20% B for 5 min, 20%–100% for 10 min and isocratic 100% B for 15 min at a flow rate of 0.4 mL/min. The sample injection volume was 20 µL, and detection was performed at 520 nm.

### 2.4 Encapsulation of Anthocyanin‐Rich Extracts From BRB

For encapsulation, purified anthocyanin extract (AN) (Section 2.2), isolated pea protein (PR, 80% protein) (Zona Cerealista, São Paulo, Brazil), and maltodextrin (MA, DE20) (Ingredients Online, São Paulo, Brazil) were used as core and wall materials. Two formulations were prepared: (i) 100% of PR (sample titled PP) and (ii) 50:50 (w/w) of PR:MA (sample titled PM), both containing 2.5% (w/w, relative to wall material) of ANC (defined by previous tests).

Initially, PR and MA were separately dispersed in citrate buffer (50 mL, 10 mM, pH 3.0) under magnetic stirring (≈1000 rpm) (IKA C‐MAG HS7 digital) at room temperature for 30 min to ensure complete hydration. For the PR:MA system, MA was first fully solubilized, followed by the gradual addition of PR to avoid aggregation. The pH of the biopolymer solutions was monitored and adjusted to 3.0 ± 0.05 using 0.1 M HCl when necessary.

Subsequently, the anthocyanin extract was slowly added dropwise to the biopolymer solutions under continuous stirring. The system was then heated to 40°C and maintained under agitation (≈1000 rpm) for 120 min to promote molecular interactions between ANC and the wall materials (Tan et al. 2024). During this period, the pH was periodically monitored to ensure stability, as slight variations may occur due to protein–polyphenol interactions. After the interaction period, the dispersions were immediately frozen at −18°C for 24 h and subsequently freeze‐dried (−55°C, 25 µHg) (LIOTOP L101, São Carlos, Brazil).

### 2.5 Encapsulation Efficiency (EE)

The encapsulation efficiency (EE) was determined according to Mansour et al. (2020). For total anthocyanin content (TAC), 200 mg of microcapsules and 1 mL of distilled water were added and vortexed for 5 min. Then, 9 mL of ethanol was added, followed by sonication for 10 min using an ultrasound probe (Eco‐Sonics, Ultronique QR550, Brazil). To determine the surface anthocyanin content (SAC), 200 mg of microcapsules was used, extracted using 10 mL of ethanol, and vortexed for 30 s. Both samples were centrifuged, and the supernatant was collected. The TMA was evaluated according to Section 2.3, and EE was calculated by Equation (3):

(3) $$\mathrm{EE}(\%)=\frac{\mathrm{TAC}-\mathrm{SAC}}{\mathrm{TAC}}\times 100\%$$

### 2.6 Particle Size and Solubility

The mean hydrodynamic particle size analysis was performed by dynamic light scattering (DLS) using a Zetasizer Nano ZS (Malvern Instruments, UK). The samples were previously diluted to achieve an optimal concentration for analysis and avoid multiple scatterings. Observations were performed at 25°C, with a detection angle set at 173° (backscatter mode), which is suitable for particles in a colloidal suspension. Each sample was tested in triplicate, with 20 runs per measurement, and the results were expressed as the mean hydrodynamic diameter (*z*‐average).

Solubility analysis was performed according to Deng et al. (2023), in which 0.1 g of capsules was added to distilled water (10 mL) at 30°C, magnetically stirred for 30 min, and then centrifuged (10 min). The resulting supernatant was dried at 105°C until constant weight, and the solubility calculation corresponds to the percentage by mass of the dry solid present in the microcapsules before drying.

### 2.7 Scanning Electron Microscopy and Confocal Laser Scanning Microscopy

The morphological characteristics of encapsulated anthocyanins were evaluated using scanning electron microscopy (SEM) JSM 6390LV (JEOL, Tokyo, Japan) coupled to a tungsten electron source, a secondary electron detector, and an accelerated voltage of 10 kV.

Confocal laser scanning microscopy (CLSM) was used to evaluate the microstructure and fluorescence behavior of anthocyanins (ANC), pea protein (PE), maltodextrin (MA), and the encapsulated systems containing only pea protein (PP) or pea protein combined with maltodextrin (PM). Each sample was individually deposited onto clean microscope glass slides and evenly dispersed to form a thin layer suitable for imaging.

The analyses were performed using a confocal laser scanning microscope (Leica TSC SP5/DMI 6000) equipped with a 20× objective lens. Excitation was carried out using a 514 nm laser line, and the emitted fluorescence was collected within the spectral range of 520–600 nm. Images were acquired under identical instrumental conditions whenever possible to enable qualitative comparison among samples of fluorescence intensity, particle morphology, and anthocyanin distribution within the encapsulating matrices.

### 2.8 Fourier Transform Infrared Spectra and Thermal Analyses

Fourier transform infrared (FTIR) spectra of encapsulated anthocyanins were recorded in a Cary 600 Series (Agilent Technologies, St. Clara, USA) in attenuated total reflectance (ATR) mode using a wavelength range of 4000–500 cm^−1^, with a 4 cm^−1^ resolution and accumulation of 16 scans.

Differential scanning calorimetry (DSC) analysis was performed in an STA 449 F3 Jupiter (Netzsch, Germany). Between 5 and 10 mg of the sample was added to the sealed aluminum capsules. The temperature increased from 25°C to 300°C at 10°C/min. The nitrogen flow rate was 20 mL/min.

### 2.9 Antioxidant Activity

The dried samples (PP and MP) were dissolved (5–10 mg/mL) in methanol and prepared using a vortex mixer. The DPPH method performed antioxidant activity analysis according to Brand‐Williams et al. (1995), FRAP according to Benzie and Strain (1996), and TCR according to Berker et al. (2013), all methods adapted for microplates.

### 2.10 Storage Stability

The encapsulated anthocyanin powder was packed in vials and stored at 25°C for 0, 15, 30, 45, and 60 days. After each time point, TMAs during storage were measured using the colorimetric method (Section 2.3). A kinetic model was fitted according to Equation (4), and the time of half‐life (*t*_(1/2)_) was determined by Equation (5):

(4) $$\frac{A_{t}}{A_{0}}=e^{-k_{d}\times t}\;$$

(5) $$t_{1/2}=\frac{\ln\left(2\right)}{k_{d}}\;$$

where $A_{t}$ is the absorbance with time *t*, $A_{0}$ is the initial absorbance, $k_{d}$ is the degradation rate (days^−1^), and *t* is the time (days).

The color characterization for each storage time was performed using a colorimeter (Delta Vista 450G, Delta Color, Brazil) with the CIELab coordinates, where L* represents the lightness index; a* represents the tonalities from green −a* to red + a* color; and b* represents the tonalities from blue −b* to yellow +b* color. The hue angle (*h*°) = tan^−1^ (*b*/*a*) and Chroma (C*) = (*a*_2_ + *b*_2_)^0.5^ were calculated as the means of three replicates in each sample. The CIELAB values were converted into images using the Nix Sensor online color converter (Nix Sensor Ltd., Hamilton, ON, Canada; available at https://www.nixsensor.com/free‐color‐converter).

### 2.11 In Vitro Release Experiment

In vitro release experiment analysis followed the protocol according to Minekus et al. (2014) and Polmann et al. (2025). For the tests, 0.5 g of sample (PP or PM) was used, undergoing three steps of digestion with α‐amylase (75 U/mL) to simulate mouth conditions (oral phase), at 37°C with shaking at 100 rpm for 2 min; digestion with pepsin (2000 U/mL) and HCl to simulate gastric conditions (gastric phase) at 37°C with shaking at 100 rpm for 2 h; and digestion with bile salts (10 mM) pancreatin (800 U/mL) and sodium bicarbonate (1 M) to simulate small intestine conditions (intestinal phase), at 37°C with shaking at 100 rpm for 2 h. Analysis of anthocyanins released during digestion was performed by HPLC–PAD (Section 2.4), and the calculation was performed based on the initial concentration of C3G (100%) to obtain the percentage of anthocyanin released.

### 2.12 Statistical Analysis

All results are presented as the mean ± standard deviation. The dataset was evaluated by *t*‐test or one‐way analysis of variance (ANOVA), followed by Tukey's test at a probability level of less than 5% (*p* < 0.05), and significant differences were determined. All statistical procedures were performed using Statistica v. 13.5 software (TIBCO Software Inc., Palo Alto, CA, USA).

## 3 Results and Discussion

The purified anthocyanin (from BRB) used for encapsulation was identified by HPLC–DAD (Figure S1). The main anthocyanin was identified as C3G, as reported by Leonarski, Cesca et al. (2025). The crude extract (before purification) had already been evaluated and found not to exhibit cytotoxicity in L929 cells at concentrations up to 1000 mg/L (Leonarski Cesca, et al. 2025). The purified extract (approximately 90% of C3G) at a concentration of 200 µM (approximately 100 mg/L) was found to be nontoxic to NCI‐N87 cells and Caco‐2:MTX‐HT29 cells for 4 h (gastrointestinal absorption time) (Leonarski, Kuasnei, et al. 2025). The capsules presented total anthocyanin levels of 0.65 ± 0.08 mg C3G/g for PP and 0.70 ± 0.03 mg C3G/g for PM, which did not differ significantly (*p* = 0.29; *p* > 0.05).

### 3.1 EE

The EE was 90.2 ± 1.39% for the sample encapsulated with pea protein (PP) and 94.7 ± 0.51% for the sample containing pea protein and maltodextrin (PM), which was a significant difference (*p* = 0.006, *p* < 0.05). The complex coacervation between the protein and maltodextrin favored the encapsulation of anthocyanins from BRB. Coacervation depends on the associated process parameters, as well as the characteristics of the wall polymers and the encapsulated compound, resulting in varying encapsulation efficiencies (Vergara et al. 2023).

To date, the use of pea protein in combination with maltodextrin has been associated with oil encapsulation to prevent the effect on oxidative stability (Kurek and Pratap‐Singh 2020; Benito‐Román et al. 2020). However, no studies have been conducted on encapsulating anthocyanins or phenolic compounds. Vergara et al. (2023) encapsulated blackberry phenolic compounds with pea protein and different gums (carrageenan, tragacanth, and xanthan), and the authors reported EE (%) values similar to our study, ranging from 89.9% for xanthan gum to 94.5% for tragacanth gum.

### 3.2 Structural and Thermal Characteristics of Microcapsules

The particle size of the obtained capsules for PP was 75.7 ± 3.7 µm, while for PM, the value was 167.9 ± 9.3 µm. These values differed significantly (*p* = 0.00009, *p* < 0.05). García‐Segovia et al. (2021) encapsulated beetroot pigments using 3.5%–7% pea protein by spray drying. Their results for particle size ranged from 34 to 94 µm. In the study performed by Souza et al. (2017), the particle size was 370.89 µm using anthocyanin‐rich extract from jaboticaba pomace encapsulated with maltodextrin. A mixture of pea protein and maltodextrin (PM) provided intermediate values of the particle size between encapsulated only protein or maltodextrin. Smaller particle sizes can lead to better solubility, with solubility being correlated to particle size, although agglomeration can also affect the solubility of microcapsules (Nthimole et al. 2022). Conversely, larger particle sizes can improve EE (Chen et al. 2023), as observed in our study.

The solubility results showed that the composition of the wall material significantly affected the capsule reconstitution properties. The PP sample showed a solubility of 72.3 ± 3.0%, while PM showed a solubility of 82.1 ± 1.3%, differing significantly (*p* = 0.017, *p* < 0.05). This improvement can be attributed to the high hydrophilicity and rapid dissolution of maltodextrin, which favors water absorption, particle wettability, and powder dispersion in aqueous medium. In addition, maltodextrin generally forms amorphous matrices with high water affinity, contributing to faster reconstitution of encapsulated bioactive compounds (Fang and Bhandari 2010; Cheng et al. 2023).

In contrast, the lower solubility observed in PP capsules may be due to stronger protein‒protein interactions, leading to denser aggregates and reduced water penetration into the matrix. Protein‐rich particles generally exhibit greater structural cohesion after drying, which can delay hydration and dissolution. Therefore, the combination of pea protein with maltodextrin (PM samples) likely yielded a less compact, more porous structure, thereby improving water diffusion and particle disintegration. Similar synergistic effects between proteins and carbohydrates have been reported for the development of powders with enhanced solubility and technological functionality (Liu et al. 2026; Song et al. 2022).

**FIGURE 1:**
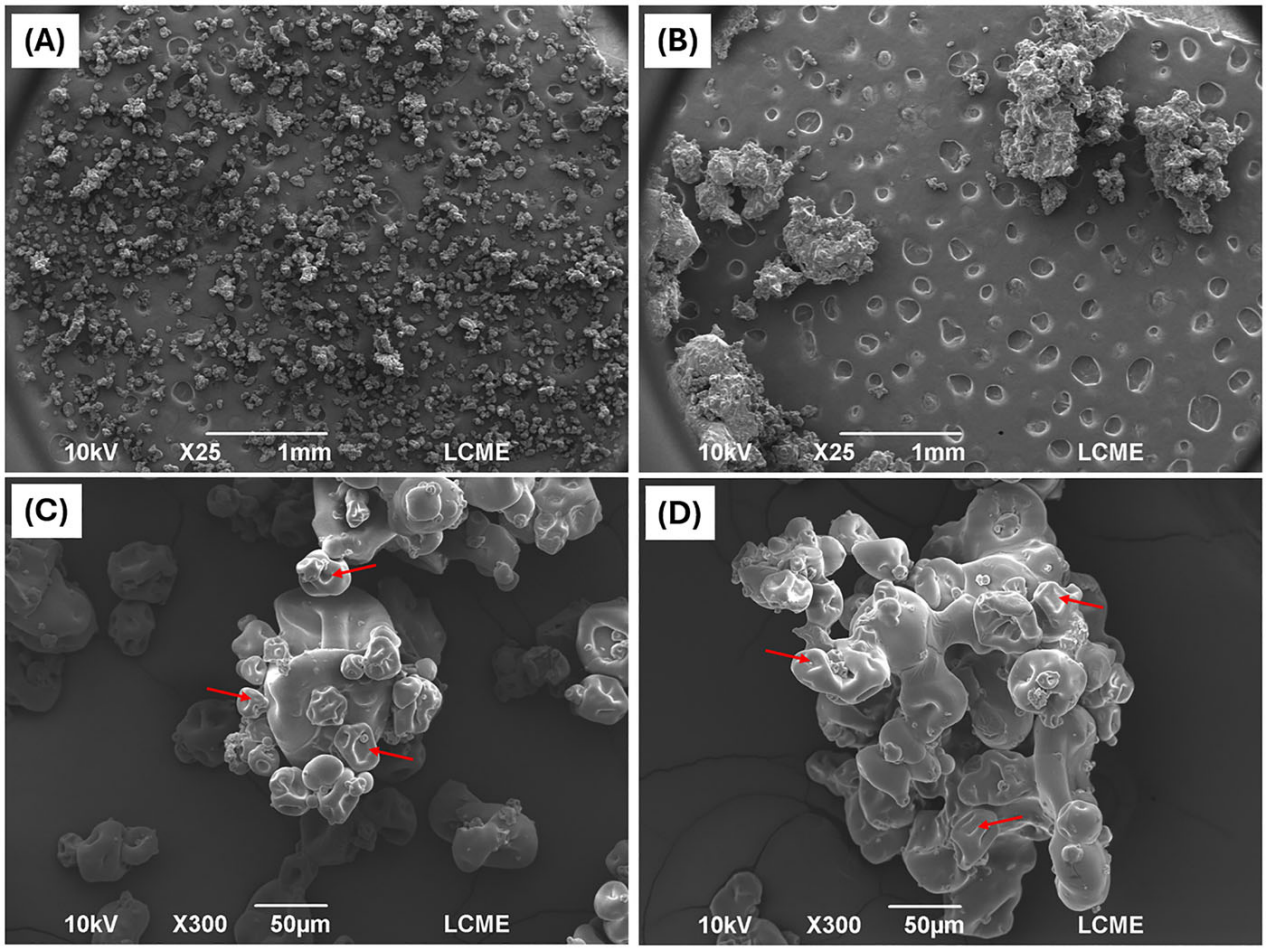
Micrography for (A and C) PP and (B and D) PM samples.

According to micrography (Figure 1) of the samples (PP and PM), the capsules were irregular, with agglomeration and cavities on the surface (some examples are indicated by red arrows), and a similar result was reported by Li et al. (2022) when using soy protein isolate as the wall material. It can be observed that for PP (Figure 1A,C), the agglomeration phenomenon was lower compared to the PM samples (Figure 1B,D). Muñoz‐Coyotecatl et al. (2025) presented similar results for encapsulated anthocyanin‐rich colorants from red cabbage encapsulated with inulin, gum arabic, and pea protein. When coacervating the protein with inulin or gum arabic, agglomeration of the capsules was observed. If microcapsules exhibit a wide size distribution, the larger particles can act as aggregation nuclei (Mohammadalinejhad and Kurek 2021), thereby hindering their solubility. Furthermore, coacervation resulted in a larger particle size (observed in Figure 3C,D), corroborating a previously reported result.

Laser confocal microscopy enabled the selective detection of anthocyanin fluorescence (*λ* = 514 nm), as proteins and polysaccharides did not exhibit significant emission at this wavelength (Figure S2). Thus, the observed signals can be directly attributed to the distribution of anthocyanins in the encapsulating matrix (Le Bourvellec and Renard 2012; Buchweitz et al. 2013).

**FIGURE 2:**
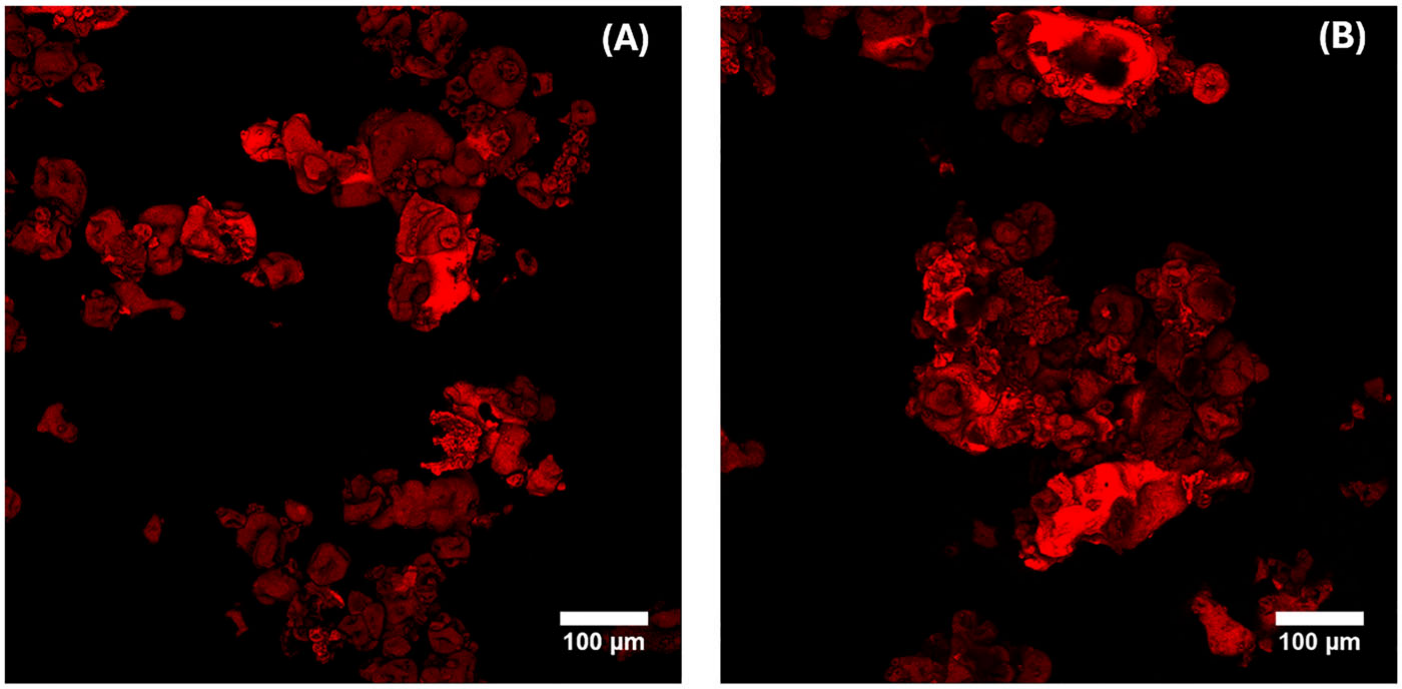
Confocal laser scanning microscopy (CLSM) images of anthocyanin microcapsules: (A) PP and (B) PM. Red fluorescence corresponds to the anthocyanin signal (*λ* = 514 nm; 520–600 nm). Images were obtained using a 20× objective lens.

In the system containing only pea protein (Figure 2A), well‐defined fluorescent structures were observed, but with a heterogeneous distribution, with regions of higher intensity interspersed with less fluorescent areas. This behavior indicates efficient incorporation (approximately 90%, as verified by EE) but is not uniform, possibly due to protein–anthocyanin interactions through hydrogen bonds and hydrophobic forces, which may favor localized aggregation (Jakobek 2015; Ozdal et al. 2013).

The system containing pea protein and maltodextrin (Figure 2B) exhibited more homogeneous, continuous fluorescence, resulting in better anthocyanin dispersion. Maltodextrin can act as a structuring agent, reducing protein aggregation and favoring the formation of a more organized and stable matrix (Dickinson 2010; Song et al. 2022). This structural effect is associated with increased EE (approximately 95%), which may improve the retention of bioactive compounds. Furthermore, maltodextrin can increase the consistency of the medium and reduce diffusion losses during the encapsulation process (Comunian et al. 2013; Laureanti et al. 2023). In addition, the more uniform distribution also suggests less surface exposure of the anthocyanins, which may favor greater protection against light, oxygen, and pH variations, factors responsible for their handling. More fluid matrices tend to exhibit greater physicochemical stability (Cheng et al. 2023; Castañeda‐Ovando et al. 2009).

**FIGURE 3:**
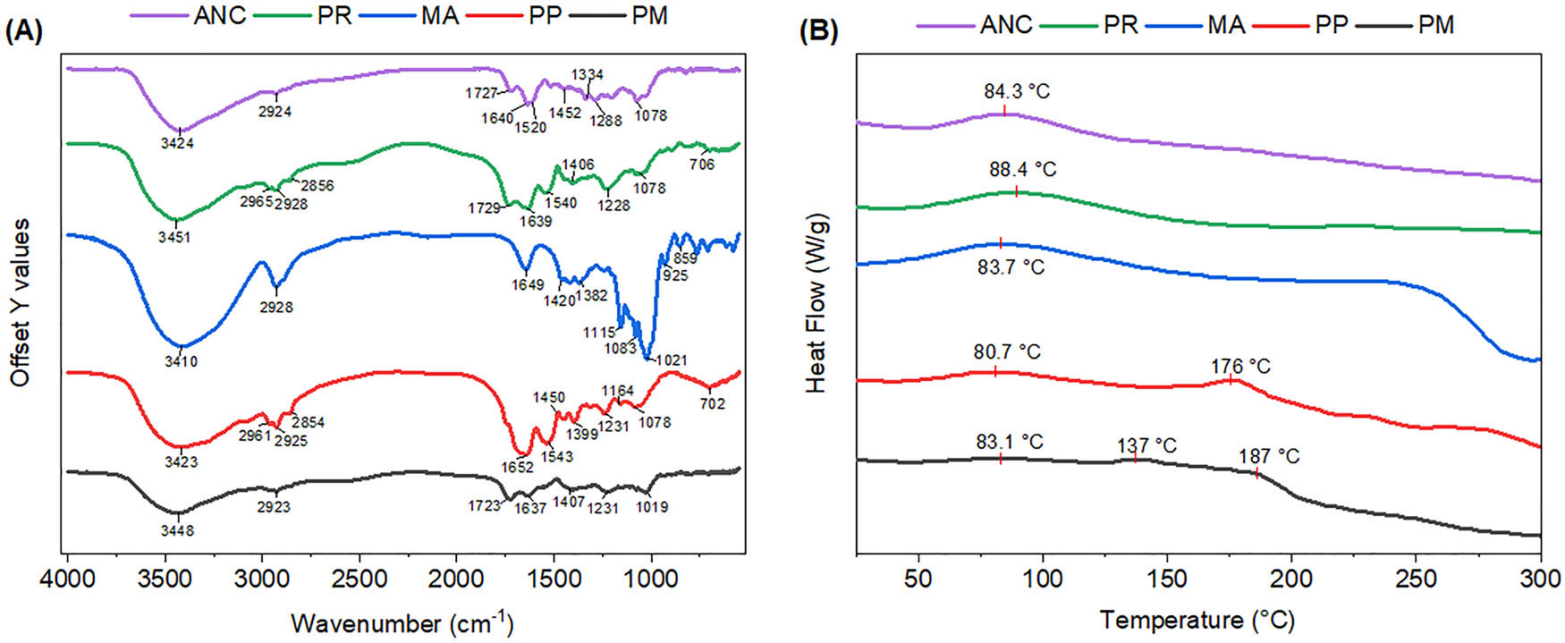
(A) FTIR spectra and (B) DSC analysis for ANC, PR, MA, and for PP and PM microcapsules.

Figure 3A presents the FTIR results obtained for both the encapsulated samples, the anthocyanin (ANC), and the wall materials used (PR and MA). A band was observed at approximately 3420 cm^−1^ for all samples, corresponding to a decrease of one hydroxyl group. A peak was also observed at approximately 2920 cm^−1^ due to the stretching vibrations of C─H_2_ and C─H_3_. The combination of ANC and PP (forming PP:ANC) showed colocalization of the C═O group and amide II (1652 and 1543 cm^−1^, respectively). A new peak at 1723 cm^−1^ (dominated by the alkyl C═O group) was present in PR:MA:ANC (possibly originating from ANC and PR). The peaks near 1250 and 1050 cm^−1^ were attributed to the stretching vibration and bending vibration of the C─O─C bond, respectively (Mansour et al. 2020; Tan et al. 2024). Both encapsulated samples exhibited shifts and lower intensities for most groups compared to the spectrum of individual compositions and interactions between ANC and PR and MA, primarily due to electrostatic interactions and hydrogen bonds (Mansour et al. 2020).

**FIGURE 4:**
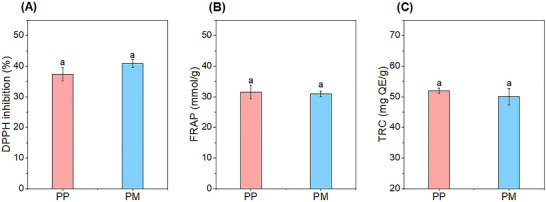
Antioxidant activity of PP and PM microcapsules evaluated by (A) DPPH inhibition, (B) FRAP, and (C) total reducing capacity (TRC). Means with different letters different significantly (*p* < 0.05).

As observed in Figure 3B, both ANC and the wall materials (PR and MA) showed an endothermic peak between 83°C and 88°C. According to Andrade et al. (2021), this first peak is related to the loss of moisture and volatile components. Salah et al. (2020) observed an endothermic peak at 82°C for anthocyanins purified from raspberry (C3Gs and cyanidin 3‐sophoroside being the main anthocyanins). For isolated pea protein (PR), the peak was approximately 88°C, according to reports by Liang et al. (2024) and Ghobadi et al. (2021), who suggested that this peak is related to the evaporation of bound water from the protein. MA was observed at approximately 84°C, and Q. Wang et al. (2024) reported an endothermic peak for MA at approximately 91°C.

Regarding the microcapsules, the first peak was less intense than the others, decreasing for PP and PM at 80.7°C and 83.1°C, respectively. The addition of ANC modified the PR structure by interacting with these compounds, making it less stable to heat treatment and lowering its denaturation temperature. This effect was also reported by Liang et al. (2024) during the preparation of pea protein isolate‐chlorogenic acid nanoparticles. The addition of MA may have aided the reorganization of these compounds (Barba‐Ostria et al. 2024), as the denaturation temperature was similar to that of the individual compounds (83°C–84°C). New peaks were observed in the microcapsules at high temperatures, which, according to Sakulnarmrat et al. (2022), indicate the formation of complexes.

### 3.3 In Vitro Antioxidant Activity of Anthocyanin Microcapsules

There was no significant difference (Figure 4) between the capsules regardless of the radicals (DPPH, FRAP, and total reducing capacity [TRC]). This is because the main component that presents antioxidant activity is anthocyanins, and these were added in the same proportion in both samples (PP and PM). The capsules exhibited inhibition of approximately 37%–40% against the DPPH radical at a concentration of 5 mg/mL. Using 10 mg/mL, the samples presented antioxidant activity by the FRAP radical of approximately 31 mmol TE/g, while for TRC, the values were approximately 51 mg QE/g.

In the study by García‐Segovia et al. (2021), the authors observed that changes in temperature (from 125°C to 150°C) during spray drying led to the degradation of PR‐encapsulated beetroot bioactive compounds, resulting in a decrease in their antioxidant capacity. However, the authors reported that increasing the PR concentration (from 3.5% to 7%) resulted in greater protection of the bioactive compounds.

### 3.4 Storage Stability of Anthocyanin Microcapsules

The effect of storage stability on color (Table 1) was evaluated by measuring color parameters at 0, 15, 30, 15, and 60 days (storage at 25°C). The PP sample presented higher luminosity (L*) compared to PM, possibly due to the amount of pea protein added. Over the course of the days, both samples lost luminosity during storage (*p* < 0.05) from 15 days and continued to do so until day 60, indicating sample discoloration. A similar effect was reported by Mansour et al. (2020), in which, after 60 days, microencapsulated red raspberry anthocyanins (using soy protein isolate and gum arabic) lost luminosity (at 25°C and 37°C).

**TABLE 1:** Color indicates (L*, a*, b*, C*, h°) in PP (pea protein) and PM (pea protein + maltodextrin) microcapsules for 60 days of storage at 25°C.

Samples	Day 0	Day 15	Day 30	Day 45	Day 60
	L*				
PP	21.51 ± 0.64^d^	25.28 ± 0.26^c^	24.94 ± 0.25^c^	27.83 ± 0.36^b^	30.35 ± 0.12^a^
PM	10.79 ± 0.46	13.53 ± 0.11^b^	13.45 ± 0.22^b^	14.43 ± 0.30^ab^	15.22 ± 0.12^a^
	a*				
PP	25.57 ± 0.54^d^	28.95 ± 0.37^b^	27.25 ± 0.34^c^	28.00 ± 0.11^bc^	30.57 ± 0.10^a^
PM	23.16 ± 0.55^a^	23.12 ± 0.26^a^	21.82 ± 0.54^a^	22.46 ± 0.32^a^	21.99 ± 0.01^a^
	b*				
PP	3.80 ± 0.17^b^	4.04 ± 0.12^b^	4.14 ± 0.11^b^	4.25 ± 0.06^b^	4.98 ± 0.12^a^
PM	5.55 ± 0.25^a^	5.30 ± 0.25^ab^	5.80 ± 0.15^a^	4.65 ± 0.12^b^	5.34 ± 0.06^a^
	C*				
PP	25.85 ± 0.56^d^	29.23 ± 0.35^b^	27.56 ± 0.29^c^	28.32 ± 0.10^bc^	30.97 ± 0.09^a^
PM	23.82 ± 0.50^a^	23.72 ± 0.29^a^	22.58 ± 0.35^a^	22.94 ± 0.34^a^	22.63 ± 0.01^a^
	*h*°				
PP	8.47 ± 0.37^ab^	7.95 ± 0.31^b^	8.64 ± 0.15^ab^	8.63 ± 0.14^ab^	9.25 ± 0.25^a^
PM	13.52 ± 0.75^ab^	12.91 ± 0.50^bc^	14.89 ± 0.28^a^	11.71 ± 0.28^c^	13.66 ± 0.14^ab^
	Color				
PP	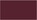	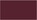	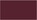	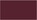	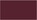
PM	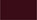	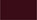	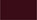	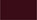	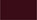

*Note*: Means with different letters in the line are significantly different (*p* < 0.05).

Regarding the a* parameter, this indicates a tendency toward red colors attributed to the high anthocyanin content (Leonarski et al. 2023). In the case of the PP sample, there was a significant increase (*p* < 0.05) in this value, indicating a greater tendency toward red during storage, with the color changing over the days observed, as shown in Table 1. For the PM sample, there was no significant difference during storage, with these values being lower compared to PP. In addition, positive b* values indicate a tendency toward yellow. A small increase was observed after 60 days for the PP sample, whereas no significant difference was noted during storage for the PM sample. This increase in the PP sample may be correlated with the wall material used (in this case, pea protein), which has a yellowish color.

Chromaticity (C*) measures the color saturation or intensity, as well as the hue angle value (°*h*°) related to the color tone. Only the PP sample showed an increase in the C* value during storage, while PM presented similar values for the C* parameter and both samples (PP and PM) for the *h*° parameter during storage. The increase in this parameter may also be related to the wall material (Mansour et al. 2020). The low *h*° value and high C* and a* (red color intensity) are possibly due to the high concentration of anthocyanins (Leonarski et al. 2023).

**FIGURE 5:**
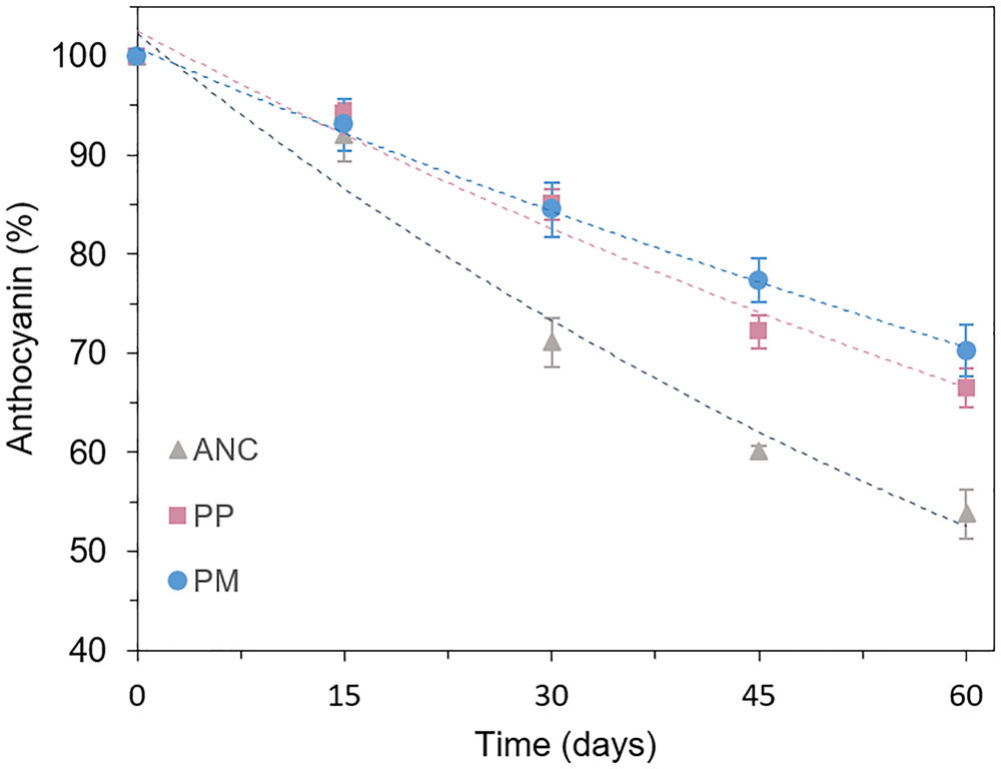
Storage stability at room temperature (∼25°C) of ANC and microcapsules (PP and PM). The dashed lines correspond to the fit of the degradation model.

During storage, from day 30 onward, ANC showed a significant difference (*p* < 0.05) from the capsules (Figure 5), while the capsules (PP and PM) showed a significant difference on day 45 (*p* < 0.05). However, at the end of 60 days, these did not show a significant difference (*p* > 0.05). In general, the capsules demonstrated superior protection after 60 days of storage, achieving an anthocyanin retention of 66%–70%, whereas the nonencapsulated anthocyanin (ANC) showed a retention of 53%.

Tan et al. (2024) found a retention of 53.8% for ANC extracted from black rice after 60 days, while samples encapsulated with pectin and carboxymethylcellulose showed retention rates between 58.7% and 85.2%. In the study performed by Mansour et al. (2020), the retention of red raspberry anthocyanin encapsulated with soy protein isolate and gum arabic as the wall material ranged from approximately 48%–60.3%. In contrast, for the control (without encapsulation), it was 39.36%.

Based on the storage stability results, the kinetic model was adjusted to estimate kinetic parameters, including the degradation rate constant (*k*) and half‐life time (*t*_1/2_). The *k* values were 0.011 (day^−1^), 0.0072 (day^−1^), and 0.0059 (day^−1^) for ANC, PM, and PP, respectively, resulting in *t*_1/2_ values of approximately 63, 96, and 117 days, respectively. The three regression models presented high regression coefficients (R^2^): 0.997 for PM, 0.974 for PP, and 0.972 for ANC. Therefore, although the degradation did not show a significant difference between PP and PM, according to the models, in the long term, PM will degrade more slowly.

Both PP and PM microcapsules exhibited similar protective effects, although the addition of maltodextrin resulted in a slightly higher protective value. This may have occurred due to hydrogen bonds that formed between pea proteins and maltodextrin during the encapsulation process, an effect also observed by Lan et al. (2019) in the spray‐drying process.

### 3.5 In Vitro Anthocyanin Release

**FIGURE 6:**
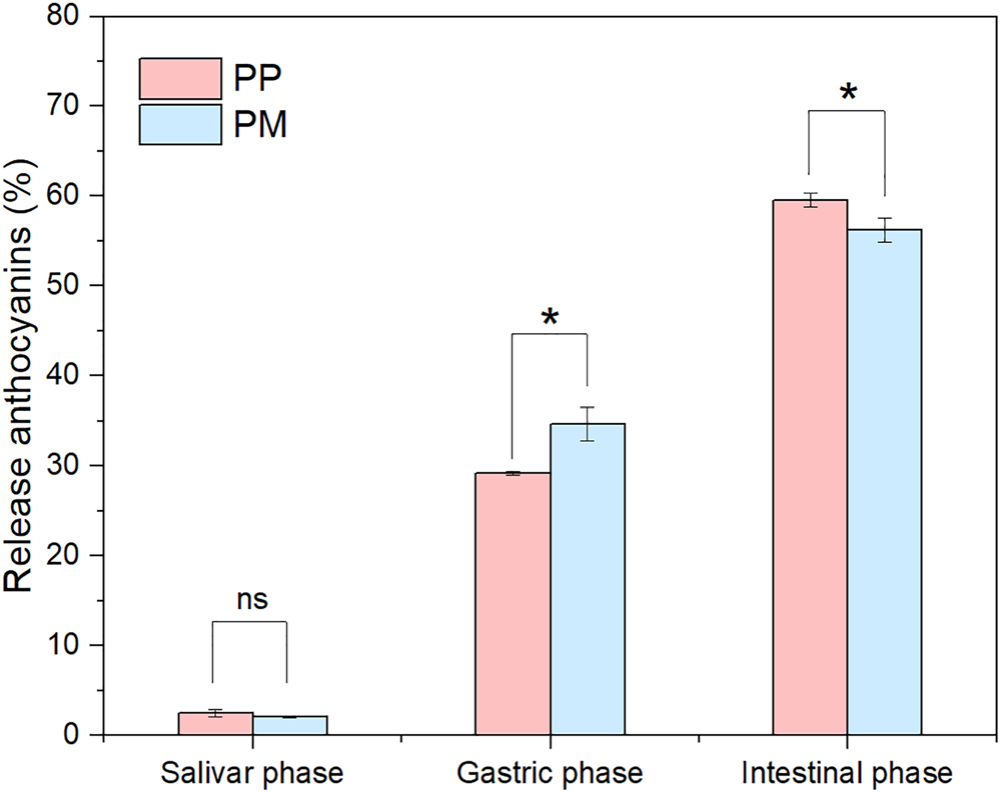
In vitro anthocyanin release of microcapsules (PP and PM) during the salivary, gastric, and intestinal phases. *Significant difference (*p* < 0.05).

Figure 6 shows the in vitro analysis of anthocyanin release from microcapsules. Approximately 2% of anthocyanins were identified in the salivary phase, possibly referring to those that were not encapsulated. In the gastric phase, 29.2% of anthocyanins were released for the PP sample, while 34.7% were released for the PM sample, differing significantly (*p* = 0.001, *p* < 0.05). Norkaew et al. (2019) encapsulated black rice anthocyanins with whey protein and maltodextrin. The authors did not find a difference in anthocyanin release in the gastric phase for the sample encapsulated only with the protein compared with the sample containing 50% protein and 50% maltodextrin.

Regarding the intestinal phase, the PP sample exhibited a higher release of anthocyanins (59.6%), whereas the PM sample showed a 56.2% release, indicating a significant difference (*p* = 0.004, *p* < 0.05). According to Cao et al. (2023), the use of simple coacervation (using only one wall material) shows promising protection of anthocyanins during the gastrointestinal simulation phases; however, complex coacervation (protein‐polysaccharide nanocomplexes) leads to greater protection of anthocyanins, causing lower bioaccessibility compared to simple coacervation.

## 4 Conclusion

BRB is an anthocyanin‐rich upcycling source, and anthocyanins can be efficiently extracted, purified, and successfully encapsulated using pea protein and maltodextrin as wall materials. The encapsulation systems developed, particularly the PM formulation (50% pea protein and 50% maltodextrin), showed high EE and favorable structural and thermal characteristics. Both formulations exhibited significant antioxidant stability and maintained approximately 66%–70% of anthocyanin content after 60 days of storage, confirming the protective effect of encapsulation. Furthermore, the in vitro digestion results revealed that anthocyanins were effectively released under gastrointestinal conditions, with PM displaying slightly higher release rates. Overall, the findings indicate that pea protein plays a crucial role in enhancing anthocyanin stability and controlled release, while maltodextrin addition improves particle morphology and encapsulation performance. Therefore, the microcapsules developed in this study have strong potential for application in functional foods, nutraceuticals, and other products that benefit from natural bioactive compounds with improved stability and bioavailability.

## Author Contributions

**Eduardo Leonarski**: writing – original draft, investigation, formal analysis, conceptualization, methodology, visualization. **Gabriela Polmann**: investigation, formal analysis. **Guilherme Dallarmi Sorita**: investigation, formal analysis. **Paulo Alexandre Durant Moraes**: investigation, formal analysis. **Karina Cesca**: investigation, formal analysis. **Débora de Oliveira**: resources, writing – review and editing, supervision, project administration, funding acquisition, conceptualization. **Acácio Antonio Ferreira Zielinski**: conceptualization, supervision, funding acquisition, methodology, project administration, writing – review and editing, resources.

## Conflicts of Interest

The authors declare no conflicts of interest.

## Supporting information




**Supplementary Figures**: jfds71184‐sup‐0001‐FigureS1‐S2.docx

## Data Availability

All data generated during the study are included in the manuscript.
