# Supplementary material for: Pea Protein and Maltodextrin‐Based Encapsulation of Anthocyanins From Black Rice Bran: Characterization, Storage Stability, and In Vitro Anthocyanin Release
Source: J Food Sci. 2026 Jun 18;91(6):e71184. doi: 10.1111/1750-3841.71184 (PMC13279847; doi:10.1111/1750-3841.71184)

**Pea Protein and Maltodextrin-based Encapsulation of Anthocyanins from Black rice bran: Characterization, Storage stability, and in vitro anthocyanin release**

**Names and e-mail addresses for all authors**

Eduardo Leonarski^1^*, eduardoleonarski@gmail.com

Gabriela Polmann^1^, gabipolmann@gmail.com

Guilherme Dallarmi Sorita^1^, guilhermedallarmi@hotmail.com

Paulo Alexandre Durant Moraes^2^, paulo.a.d.moraes@ufsc.br

Karina Cesca^1^, karinacesca@gmail.com

Débora de Oliveira^1^, debora.oliveira@ufsc.br

Acácio Antonio Ferreira Zielinski^1^, acacio.zielinski@ufsc.br

**Author affiliations**

^1^Department of Chemical Engineering and Food Engineering, Federal University of Santa Catarina (UFSC), Florianópolis, Santa Catarina, Brazil.

^2^Department of Chemistry, Federal University of Santa Catarina (UFSC), Florianópolis, Santa Catarina, Brazil.

**Contact information for Corresponding author**

*Eduardo Leonarski

E-mail address: eduardoleonarski@gmail.com

Full postal address: Department of Chemical Engineering and Food Engineering (EQA), Campus Trindade, 88010-970 Florianópolis, SC, Brazil

**SUPPLEMENTARY MATERIAL**

**Figure S1**. C3G after purification, identified in HPLC-PAD.


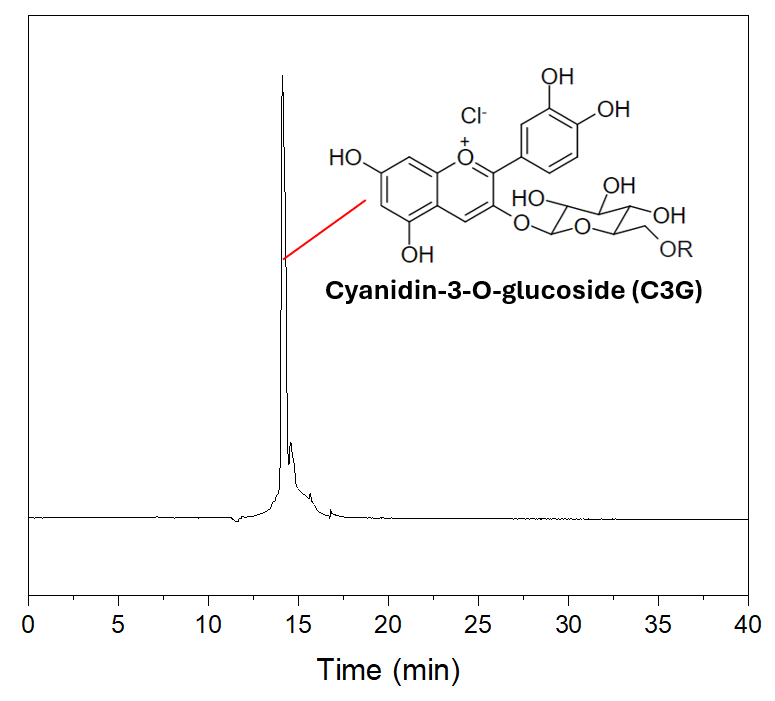


**Figure S2.** Confocal Laser Scanning Microscopy (CLSM) of anthocyanins (A), pea protein (B), and maltodextrin (C).


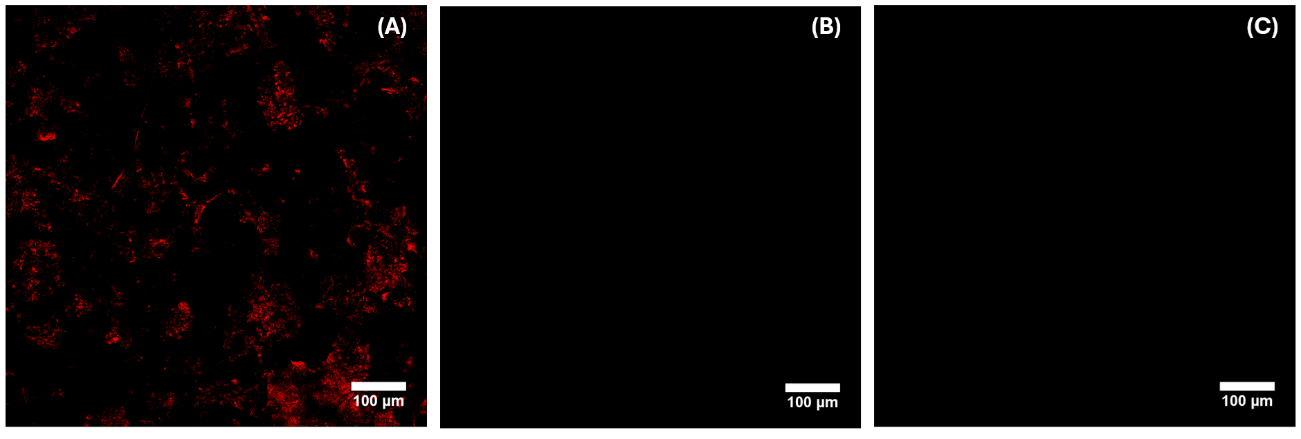

Supplement: Supplementary file 1 — Supplementary Figures: jfds71184‐sup‐0001‐FigureS1‐S2.docx [file JFDS-91-0-s001.docx]
